# Supplementary material for: Moxifloxacin Liposomes: Effect of Liposome Preparation Method on Physicochemical Properties and Antimicrobial Activity against Staphylococcus epidermidis
Source: Pharmaceutics. 2022 Feb 7;14(2):370. doi: 10.3390/pharmaceutics14020370 (PMC8875207; doi:10.3390/pharmaceutics14020370)
Supplement: Supplementary file 1 [file pharmaceutics-14-00370-s001.zip › pharmaceutics-1570351/Figure S1.pdf]

Supplementary Data for Paper *Moxifloxacin liposomes: Effect of liposome preparation method on physicochemical properties and antimicrobial activity*

Figure S1. Optimization of MOX remote loading in PC/Chol (1:1) liposomes

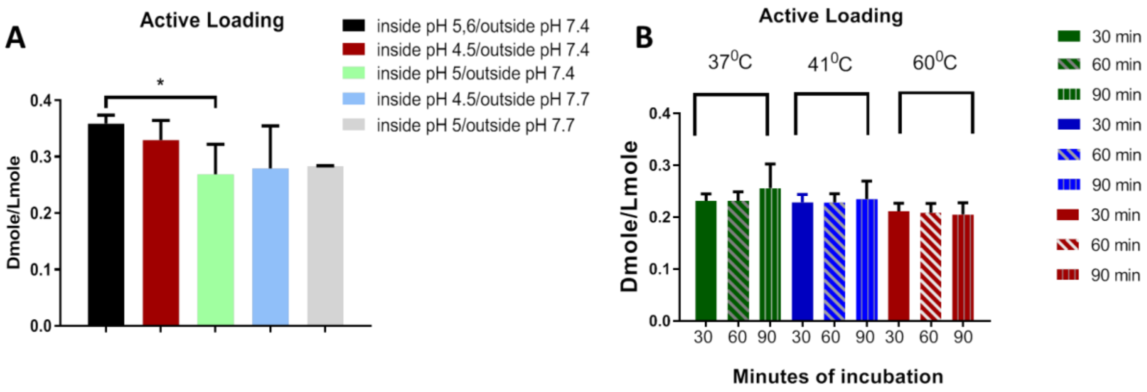

As seen from Figure S1A, the pH conditions that confer maximum loading of MOX in liposomes is $(NH_4)_2SO_4$ pH 5.60 inside and PBS, pH 7.40 outside. MOX loading is not different in the different temperatures and incubation periods tested (37, 41 or 60°C, for 30, 60 or 90 min), as seen in the results presented in Fig.S1B.
