# Supplementary material for: Moxifloxacin Liposomes: Effect of Liposome Preparation Method on Physicochemical Properties and Antimicrobial Activity against Staphylococcus epidermidis
Source: Pharmaceutics. 2022 Feb 7;14(2):370. doi: 10.3390/pharmaceutics14020370 (PMC8875207; doi:10.3390/pharmaceutics14020370)

Supplementary Data for Paper ***Moxifloxacin liposomes: Effect of liposome preparation method on physicochemical properties and antimicrobial activity***

Figure S2. Stability of liposomes during storage at 4°C : A) Vesicle mean diameter measurements; B) Polydispersity Index (PDI) measurements; C) ζ-potential values of the liposomes; D) Retention of MOX in various liposome types (lipid membrane composition).

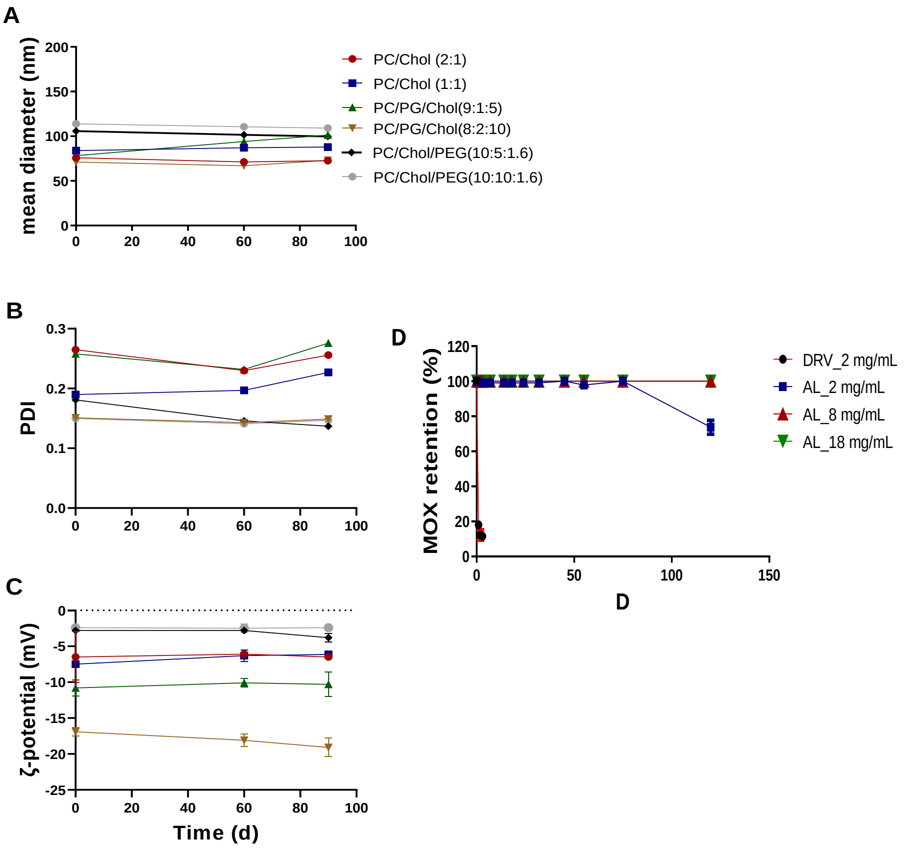

Supplement: Supplementary file 1 [file pharmaceutics-14-00370-s001.zip › pharmaceutics-1570351/Figure S2.pdf]
