# Supplementary material for: Moxifloxacin Liposomes: Effect of Liposome Preparation Method on Physicochemical Properties and Antimicrobial Activity against Staphylococcus epidermidis
Source: Pharmaceutics. 2022 Feb 7;14(2):370. doi: 10.3390/pharmaceutics14020370 (PMC8875207; doi:10.3390/pharmaceutics14020370)
Supplement: Supplementary file 1 [file pharmaceutics-14-00370-s001.zip › pharmaceutics-1570351/Figure S3.pdf]

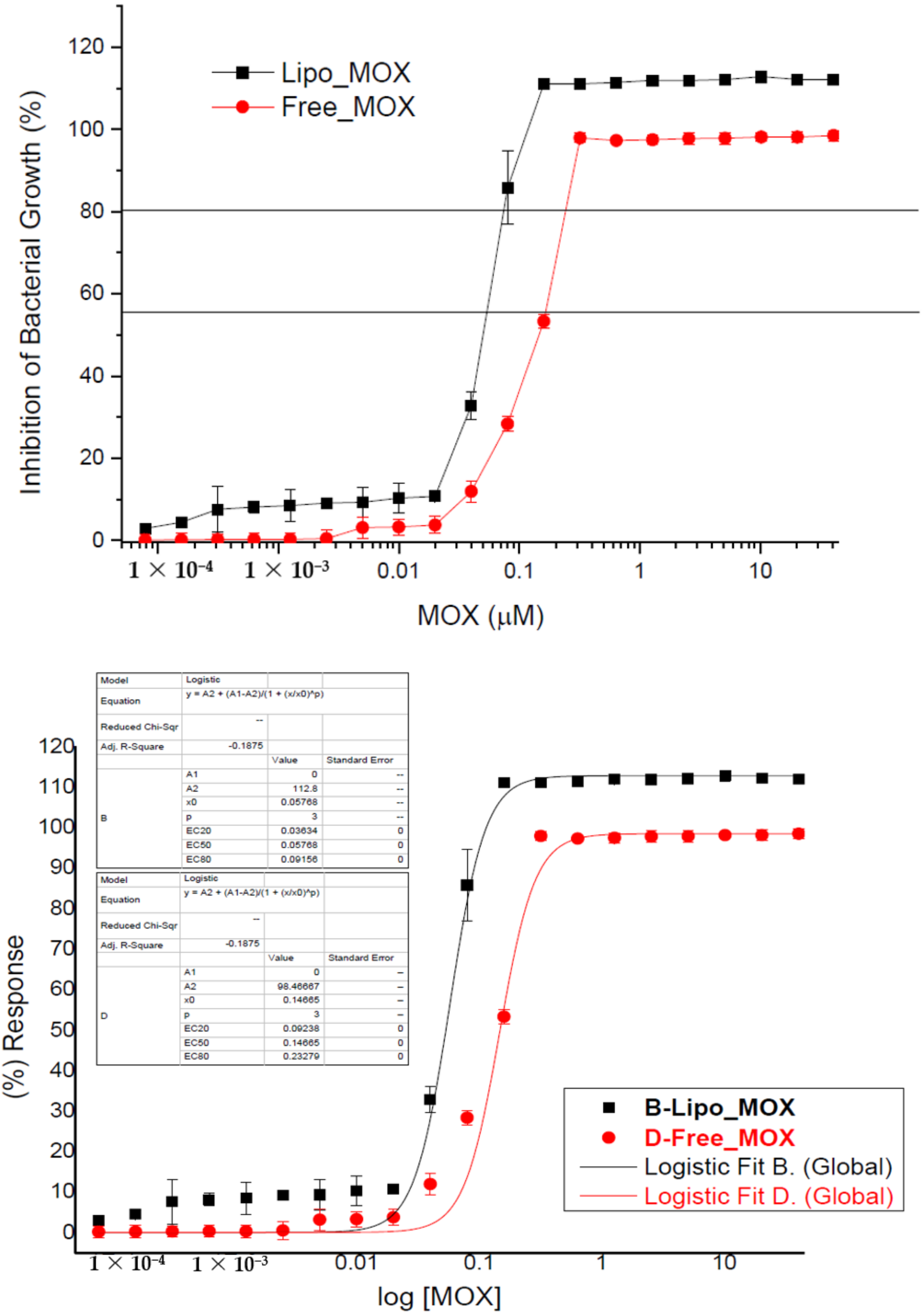

Model	Logistic		
Equation	y = A2 + (A1-A2)/(1 + (x/x0)^p)		
Reduced Chi-Sqr	--		
Adj. R-Square	-0.1875		
		Value	Standard Error
B	A1	0	--
	A2	112.8	--
	x0	0.05768	--
	p	3	--
	EC20	0.03634	0
	EC50	0.05768	0
	EC80	0.09156	0

Model	Logistic		
Equation	y = A2 + (A1-A2)/(1 + (x/x0)^p)		
Reduced Chi-Sqr	--		
Adj. R-Square	-0.1875		
		Value	Standard Error
D	A1	0	–
	A2	98.46667	–
	x0	0.14665	–
	p	3	–
	EC20	0.09238	0
	EC50	0.14665	0
	EC80	0.23279	0

Figure S3. Calculation of MIC and MIC-50 of MOX towards *Staphylococcus epidermidis* (biofilm positive, ATCC 35984) (A) Graphical estimation. (B) Logistic Calculation.
